# Systematic review of the effect of training interventions on the skills of health professionals in promoting health behaviour, with meta-analysis of subsequent effects on patient health behaviours

**DOI:** 10.1186/s12913-020-05420-1

**Published:** 2020-06-29

**Authors:** Thomas G. Hatfield, Thomas M. Withers, Colin J. Greaves

**Affiliations:** School of Sport, Exercise and Rehabilitation Sciences, University of Birmingham, Birmingham, B15 2TT UK

**Keywords:** Healthcare professional, Training, Delivery quality

## Abstract

**Background:**

We aimed to identify, synthesise and evaluate randomised control trial evidence on the effects of healthcare professional training on the delivery quality of health behaviour change interventions and, subsequently, on patient health behaviours.

**Methods:**

Systematic review with narrative synthesis of effects on delivery quality and meta-analysis of health behaviour outcomes. We searched: Medline, EMBASE, PsychInfo, AMED, CINAHL Plus and the Cochrane Central Register of Control Trials up to March 2019. Studies were included if they were in English and included intervention delivery quality as an outcome. The systematic review was registered on PROSPERO (registration: CRD42019124502).

**Results:**

Twelve-studies were identified as suitable for inclusion. All studies were judged as being high risk of bias with respect to training quality outcomes. However with respect to behavioural outcomes, only two of the six studies included in the meta-analysis had a high risk and four had some concerns. Educational elements (e.g. presentations) were used in all studies and nine included additional practical learning tasks. In eight studies reporting delivery quality, 54% of healthcare professional communication outcomes and 55% of content delivery outcomes improved in the intervention arm compared to controls. Training that included both educational and practical elements tended to be more effective. Meta-analysis of patient health behavioural outcomes in six-studies found significant improvements (Standardised mean difference (SMD): 0.20, 95% confidence interval: 0.11 to 0.28, *P* < 0.0001, I^2^ = 0%). No significant difference was found between short (≤6-months) and long-term (> 6-months) outcomes (SMD: 0.25 vs 0.15; *P* = 0.31).

**Conclusions:**

Delivery quality of health behaviour change interventions appears to improve following training and consequently to improve health behaviours. Future studies should develop more concise /integrated measures of delivery quality and develop optimal methods of training delivery.

## Background

Health impairing behaviours, such as smoking, over-eating and prolonged sedentary time are the underlying cause of many diseases (90% of cancers [1] and 30% of cardiovascular diseases [2]), a lower quality of life and a proportion of premature deaths [3]. A meta-analysis of two-cross-sectional and 16-prospective studies, comparing the impact of high and low sedentary activity, found that increased sedentary time doubles the risk of developing type 2 diabetes (relative risk = 2.1; 95%CI:, 1.6–2.8) [4]. Health impairing behaviours and their resulting diseases also place an increased financial burden on healthcare delivery. Smoking and alcohol related behaviours alone cost the NHS £5.4 billion per year [3].

The effectiveness of interventions to change health impairing behaviours has been shown to vary [5]. For example, a 22-study meta-analysis assessing the effectiveness of weight loss interventions to prevent type-two diabetes highlighted a wide range in mean weight loss (− 6.97 kg to 0.49 kg, I^2^ = 93.3%) [6]. Some interventions that appear to be effective in individual trials appear to be ineffective in meta-analyses [7].

A fundamental issue for effectiveness in behavioural interventions is quality of delivery [8, 9]. Delivery of the intended intervention content [6, 10] and communication skills [11] are two parameters of delivery quality that can influence the effectiveness of health behaviour change interventions [12]. With regard to content, the presence of specific elements have been shown to improve effectiveness. For example, a meta-analysis of 194 HIV-prevention interventions, found the presence of audio-visual media significantly increased the effectiveness of a behavioural skills intervention (homogeneity coefficient: 45.42 (96.21 with media vs 41.22 without), *P* < 0.001) [10]. With regard to communication skills, a meta-analysis of six-RCTs highlighted that different communication techniques within similar face-to-face health interventions can impact effectiveness (SMD: 0.49; 95% CI 0.02–0.96) [13].

Despite knowledge of the importance of delivery quality, systematic reviews have highlighted the need to improve intervention fidelity [14]. A recent review of 22-RCTs assessing group-based self-management interventions for osteoarthritis, highlighted that mean intervention fidelity (delivery quality) scores were low, (35%) and highly variable (range: 10–80%) [15]. Similarly, an RCT assessing three-healthcare professionals on their delivery of physical activity and smoking cessation interventions, showed wide variability in competency (mean scores of 2.9, 2.2 and 2.4, where acceptable delivery quality was defined as 3.0 or more) [16]. This variability in healthcare professional communication skills and content delivery may be a key reason underlying the variable effectiveness of behaviour change interventions [14–16].

To improve fidelity and consistency (of communication and content delivery) when delivering interventions, and ultimately their effectiveness, many studies and current NICE guidelines advise high quality healthcare professional training [5, 14–16]. However, to our knowledge, no systematic review has been undertaken assessing the overall impact of attempts to improve healthcare professional training on the quality of intervention delivery. The primary aim of this systematic review is to identify, synthesise and evaluate the effect of additional training of healthcare professionals on delivery quality, in the context of delivering health behaviour change interventions. A secondary aim is to evaluate the consequent effect of healthcare professional training on patient health behaviours.

## Methods

This systematic review and meta-analysis followed the methods outlined by the Cochrane handbook for systematic reviews [17] and was registered with Prospero (Reference number: CRD42019124502).

### Data sources and search strategy

An electronic search was performed up until 19th March 2019 using; Medline, EMBASE, PsychInfo, AMED, CINAHL Plus and the Cochrane Central Register of Control Trials, for studies published in English. The search terms used, were informed by past reviews [8, 16] and the search strategy is shown in Supplementary Table 1.

### Eligibility criteria

The training intervention must be delivered to any healthcare professional. For this systematic review a healthcare professional is a role that involves contact with patients and delivery of care and or treatment, which includes but not limited to: physicians, surgeons, nurses, physiotherapists and healthcare assistants. Included studies must assess quality of intervention delivery and be a randomised control trial.

Studies that only train participants who have contact with patients but do not deliver any care or treatment were excluded from this study, for example; hospital porter or ward clerk. Along with studies: (1) not assessing quality of intervention delivery; (2) not including a minimal or no-intervention control group; (3) not undertaking an RCT; and (4) not published in English.

### Study selection

Following duplicate removal, titles and abstracts were independently screened by two reviewers (TH, TW) (50% each), reviewers subsequently checked each other’s included studies and 20% of the excluded studies for agreement. The full texts of all potentially eligible studies were then screened independently by two reviewers (TH, TW), with disagreements resolved via discussion. Inter-rater reliability was calculated using the AC1 statistic [18]. Reference lists of similar systematic reviews found during the search and reference lists of included studies were screened for potentially eligible studies.

### Data extraction

Two reviewers (TH, TW) independently extracted data from half of the included studies, with the other reviewer checking data extraction. The following characteristics were extracted: (1) methodology (design, aim, analysis, setting); (2) participants (healthcare professional and patient: type, sample size, age, gender and qualification of trainer); (3) training interventions (duration and type of intervention, control group); and (4) outcomes (healthcare professional delivery quality (communication skills and delivery of intended intervention content), patient health behaviour change, follow-up times).

### Assessment of study quality

Study quality was independently assessed by two reviewers (TH, TW) using the Cochrane risk-of-bias tool [19], with discussion to resolve any disagreements. This standardised tool was adapted slightly to make it relevant for this systematic review. Specifically, questions relating to participant blinding (2.1 to 2.5 within domain-2a, 2.1 to 2.3 within domain-2b) were removed as blinding to training is not possible. For question 3.1 (“were all outcomes available for all participants?”) we set a criterion such that dropout rates of < 20% were answered “yes”, rates between 20 and 30% were answered “probably no”, and rates > 30% were answered “no”. When scoring individual domains, the suggested mapping and algorithms were used [19].

### Analysis

A narrative synthesis of influences of training on delivery quality was performed. As part of this, for each study we calculated the percentage of delivery quality outcomes that significantly improved compared to control. We defined short-, medium- and long-term timeframes as <six-months, ≥six to < 12-months and ≥ 12-months respectively.

To analyse health behaviour outcomes, we performed a random effects meta-analysis in Cochrane Review Manager version 5.3, using standardised mean differences (SMD) to pool effect sizes. We included data in the meta-analysis that related to the primary health behaviour targeted by the intervention. In studies where health behaviours were not reported, but other outcomes were reported that would change following the targeted behaviour (e.g. blood pressure), these outcomes were used. Behaviour change outcomes were collated at two follow up times (short-term: six-months or less and long-term: over six-months). Publication bias was not assessed due to the limited number of included studies.

## Results

### Search results

**Fig. 1:**
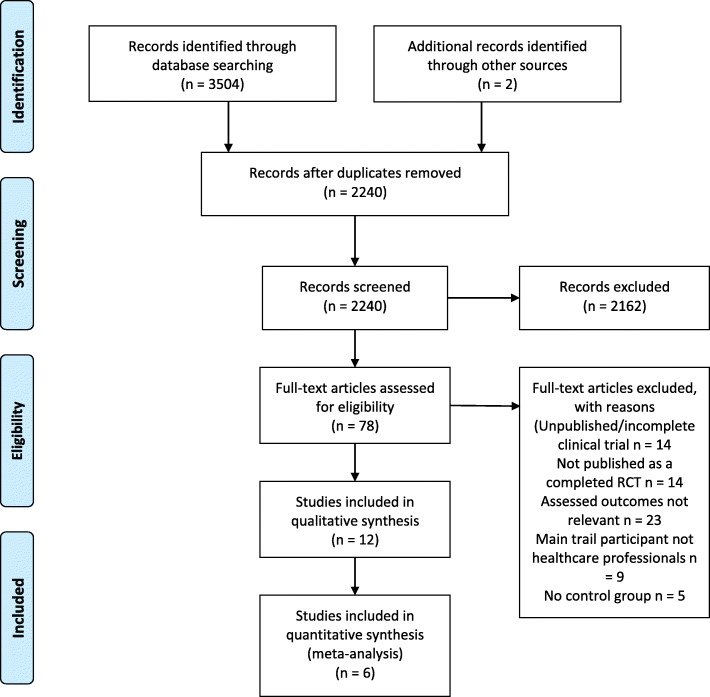
Flow-chart of search results

The search results and screening process is shown in Fig. 1. The searches returned a total of 2240 results after duplicate removal, 78-studies were reviewed at full text and 12 studies were suitable for inclusion in this review [18, 20–30]. Six studies [20, 22, 23, 25, 26, 28] were included in the meta-analysis of behavioural outcomes. The inter-rater reliability for both title and abstract and full-text screening was excellent (AC1 = 0.97 and 0.84, respectively).

### Characteristics of included studies

The characteristics and outcomes of included studies are detailed in supplementary Table 2. All training included educational elements (e.g. presentations and workshops); additional practical elements (e.g. providing opportunities to practice techniques learnt) were also included within nine studies [18, 20, 22, 25–30]. Face-to-face training was used most commonly, however online e-learning was provided in two-studies [24, 27] while one study used an audio-conference [21]. Eight-studies performed cluster sampling while five used stratified cluster sampling; five were performed in USA/Canada, three in the UK, two in the Netherlands, one in Brazil and one in Egypt. Regarding delivery quality outcomes, eight-studies reported changes in communication skills while seven reported changes in the delivery of intervention content (content-delivery). Outcomes were assessed either short-term [18, 20, 21, 26, 27] (median: one-month; range: instantaneous assessment to three-months), medium-term [20, 24, 25, 29] (median: six-months) or long-term [22, 23, 28, 30], (median: 12-months; range: 12-months to 48-months).

Regarding health behaviour outcomes, eight-studies provided patient level data, with only six studies providing data suitable for meta-analysis. The six-studies assessed a range of health behaviours, two studies examined diabetes self-care, the remaining studies examined four different health behaviours; regularity of breast feeding, smoking cessation, asthma self-care and hypertension management. Three of these studies [20, 25, 26] reported short-term outcomes (median: six-months; range: one to six-months) and three [22, 23, 28] reporting longer-term outcomes (all 12 months).

### Study quality

Risk of bias assessement results are highlighted in Tables 1 and 2. All studies reported a high risk of bias with respect to training quality outcomes, mainly due to the high number of outcome measures. However when considering only the behavioural outcomes for the six studies that were included in the meta-analysis, two had a high risk and four had some concerns.

**Table 1:**
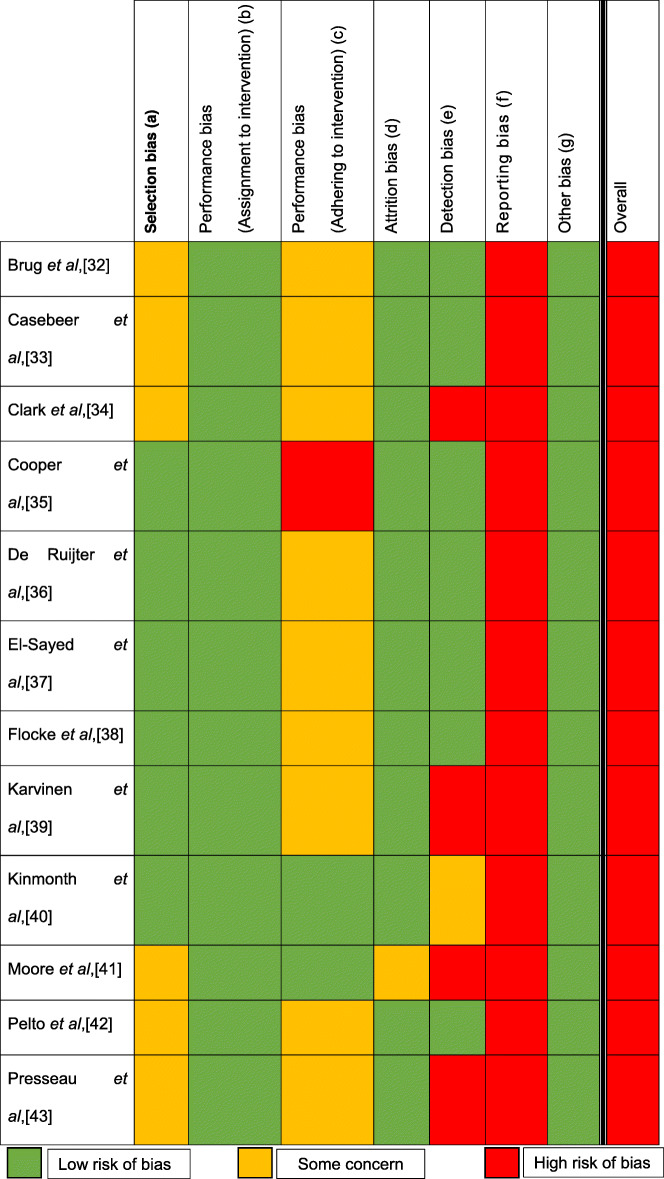
Adapted Risk of bias summary

a) Extent participants were randomly allocated to groups how this was concealed throughout enrolment (Random sequence generation, Allocation concealment)

b and c) Extent to which the researchers/participants were blinded to the group they are allocated to.

d) Assessment of the completeness of outcome data

e) Extent the outcome assessors were blinded to the intervention/control

f) Measurement of how selective researchers have been when reporting outcomes (Selective reporting)

g) Any forms of bias not covered in the five domains (Research quality)

**Table 2:**
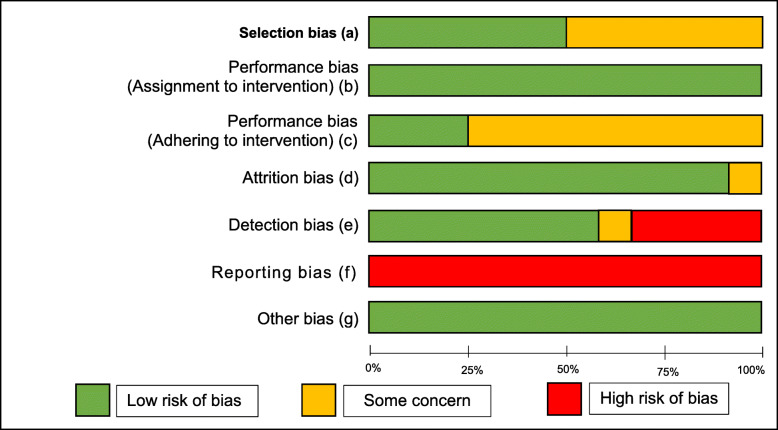
Adapted risk of bias table

a) Extent participants were randomly allocated to groups how this was concealed throughout enrolment (Random sequence generation, Allocation concealment)

b and c) Extent to which the researchers/participants were blinded to the group they are allocated to

d) Assessment of the completeness of outcome data

e) Extent the outcome assessors were blinded to the intervention/control

f) Measurement of how selective researchers have been when reporting outcomes (Selective reporting)

g) Any forms of bias not covered in the five domains (Research quality)

In summary, the high overall risk of bias was heavily influenced by the high risk of reporting bias found in all 12 studies whereby multiple outcome measures were used to assess delivery quality (a mean 11 outcome measures were used per study), with little consideration for statistical correction [31]. As well as this major source of bias, other sources were also identified. Specifically, six studies (50%) failed to report the allocation sequence or its concealment in detail, causing some concern towards their selection bias. ‘Some concern’ regarding attrition bias was recorded for one study (8%), due to a moderate drop-out rate (23.8%). When analysing detection bias, four-studies (33%) presented a high-bias risk due to use of self-report and patient recall, while one-study (8%) provided some concern due to limited blinding of assessors.

### Narrative synthesis

Within the 12 included studies a total of 132-outcomes were identified. Sixty-eight outcomes from eight-studies related to communication skills, 64-outcomes from seven-studies related to delivery of intended intervention content. Communication skills and delivery of intervention content improved significantly for 54% (37 of 68) and 55% (35 of 64) of outcomes respectively.

Two main types of training intervention were observed. These were communication training (training specifically aimed at communication skills) and content-related training (training in the delivery of specific intervention content), produced similar results, significantly improving 50 and 49% of outcomes, respectively. Training using both educational and practical elements seemed to be more effective, significantly improving 52% of outcomes, whereas education only training significantly improved only 23% of outcomes. Effectiveness was also highest in the medium-term (between six to 12-months) with significant improvements in 81% of communication and 66% of content-delivery outcomes), compared with the long-term (over 12-months), where significant improvements were found in 50% of communication outcomes and 42% of content-delivery outcomes.

### Effect on communication skills short-term

Across the three-studies [18, 20, 26] that assessed the effect of healthcare professional training on communication skills short-term, 38% of outcomes significantly improved. All three training interventions included educational and practical elements, however the two training interventions [20, 26] that specifically focused on communication skills provided the greatest benefit. Of these two-studies, “teachable moment” communication training [26], significantly improved 90% (nine of ten) outcomes measured. However, these data only relate to the delivery of smoking cessation interventions. Moreover, the limited effect of 0% (zero of six measures improving versus controls) within the other study [18] could be explained by high detection bias (use of patient recall).

### Effect on communication skills medium-term

Across the three studies [20, 25, 29] that assessed communication skills in the medium-term 81% of outcomes significantly improved. All of the interventions included educational and practical elements. Assessment of effectiveness short-to-medium term was performed by one study [20] and showed communication outcomes improved (25% vs 58%).

### Effect on communication skills long-term

Three studies [22, 23, 28] assessed effect on communication skills in the long-term, 50% of outcomes significantly improved, a decrease compared to medium-term. The least effective training intervention by this measure [23] in the medium term (which showed improvement in 25% (two of eight) of outcomes) only provided educational elements. The most effective training intervention [22], recording an improvement in 75% (six of nine) of outcomes, provided both educational and practical elements. Additionally this study used a large sample size, 74 Paediatricians, and a validated communications skills questionnaire [22].

### Effect on delivery of intervention content short-term

Three-studies [18, 21, 27], were identified as assessing the effect on delivery of intervention content in the short-term. Across these studies, 44% of the assessed outcomes significantly improved. The two [18, 27], that encompassed educational and practical activities (e.g. evaluating case studies, quizzes) were the most effective, significantly improving 40% (two of five) and 67% (two of three) of outcomes, respectively.

### Effect on delivery of intervention content medium-term

Across the three-studies [24, 25, 29], that assessed the medium-term effect of healthcare professional training on delivery of intervention content, 66% of outcomes significantly improved. The two most effective training interventions [25, 29], which improved 79% (22 of 28) and 100% (two of two) of outcomes, respectively, included both educational and practical elements.

### Effect on delivery of intervention content long-term

Across the two-studies [22, 30] that assessed the effect of healthcare professional training on intervention content-delivery in the long-term, 42% of outcomes significantly improved. Both training interventions included educational and practical elements.

### Meta-anaysis of health behaviour change

**Fig. 2:**
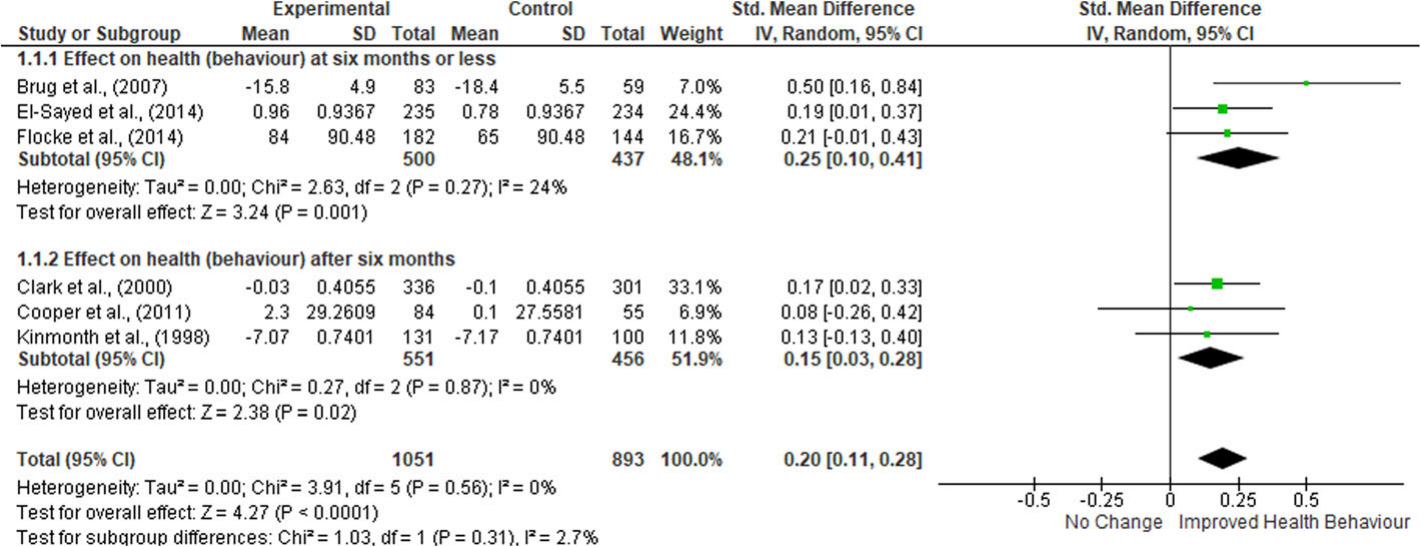
Meta-analysis of patient health outcomes

A total of six studies, with 2802 participants, were included in the meta-analysis. The results are shown in Fig. 2.

The analysis indicated that healthcare professional training resulted in a small significant improvement in patient health behaviour compared to control (SMD: 0.20; 95% CI 0.11 to 0.28; *P* < 0.01, I^2^ = 0%). Sub-group analysis highlighted a greater effect in the short-term (median six-months) (SMD: 0.25; 95% CI 0.10 to 0.41; *P* = 0.001; I^2^ = 24%) compared to the long-term (median 12 months) (SMD: 0.15; 95% CI 0.03 to 0.28; *P* = 0.02, I^2^ = 0%), however this difference was not significant (*P* = 0.31).

## Discussion

Our review suggests that healthcare professional training leads to significant improvements in healthcare professional delivery quality in the medium term (6–12 months), although this seems to decrease at 12-months. The latter finding should be interpreted with caution due to only three studies reporting outcomes at 12 months. Healthcare professional training also appears to have a significant effect on health behaviour change for up to 12 months.

These results support current NICE guidelines that state high-quality healthcare professional training is important to improve patient outcomes in health behaviour change interventions [32]. In addition to this, a review [33] of 81 RCTs of interventions to improve physicians’ practice found a small difference of 6% (interquartile range 1.8–15.6%; *P* < 0.05) in compliance with desired practice compared to the control group. This review, like ours, concluded that the inclusion of both practical and educational elements within healthcare professional training is associated with improvement in health behaviour change at the patient level.

### Strengths and limitations

This review is the first to evaluate the effectiveness of training interventions for health behaviour change in healthcare professionals. We used rigorous systematic reviewing methods [17], including second-coding of screening decisions and data extraction and we assessed the risk-of-bias of the included studies. The assessment of healthcare professional training on patient behaviour change, via a meta-analysis is also a strength, increasing precision and generalisability of results [34]. However, there are a number of limitations that need to be acknowledged. With regard to the narrative synthesis of effects on intervention delivery quality we identified a high risk of bias due to the high number of outcomes assessed and heterogeneity in the methods used and patient groups being studied. There was also noticeable heterogeneity in outcome measures, which may be due to a lack of ‘gold standard’ methods of measurement. Whilst validated and reliable methods do exist for patient-centred communication skills [35–37], there is a lack of validated measures for content-delivery in health behaviour change interventions. In the meta-analysis, the majority of the included patient outcome measures relied on patient self-report which is associated with a high risk of recall bias [38]. The comparison of short and longer-term outcomes is also limited by the relatively low numbers of studies available in each sub-group.

### Implications for clinicians and policymakers

This review supports the current NICE recommendations on the importance of healthcare professional training to enhance the effectiveness of health behaviour change interventions [32]. Policymakers should note however that the use of educational components (e.g. lectures, information-giving) alone, are unlikely to provide significant improvements in healthcare professional delivery quality. Training should also include practical (e.g. case study evaluation, practicing taught elements) elements to foster skills development and a higher level of engagement of trainees [39]. Regarding the effect of healthcare professional training on patient’s health behaviours, the finding of a small (SMD = 0.2) but significant improvement, should encourage clinicians that performing training will be beneficial to their patients. It should be noted that, although the impact on patient behaviours was relatively small, interventions aimed at healthcare professionals have the potential to be very economically efficient due to the benefits of the intervention being received by all the patients they treat. A small investment in training could impact on a large number of patients.

### Future research

There is a need for further research in this area to increase the number of high-quality trials of effectiveness of additional training to improve the quality of delivery of health behaviour change interventions. This should include research on the long-term effectiveness of healthcare professional training on both delivery quality and (objectively measured) health behaviour outcomes.

In addition to the above and to help overcome the current lack of clarity of healthcare professional delivery quality outcomes [8], it is suggested that future studies develop a standardised method that can assess delivery quality via a maximum of two scores (one for communication and one for delivery of intended intervention content). This will decrease the potential for bias due to multiple outcome measurement [40]. We also suggest that reporting of future studies complies with recognised guidelines [40]  to ensure clarity of methodology and results.

## Conclusions

This systematic review suggests that healthcare professional training is an effective tool for improving the quality of delivery of health behaviour change interventions and that providing such training improves patient health behaviours. The data also suggest that the use of both educational and practical elements seem to be key for effective healthcare professional training. The effect on delivery quality seems to last for up to 12 months but the longer-term effects are uncertain. The findings of this systematic review with respect to delivery quality should be interpreted with caution due to the high risk of bias identified in the studies reviewed. With respect to effects on behaviour change, the findings are more robust, as the studies in meta-analysis had a lower risk of bias.

## Supplementary information

**Additional file 1: Table S1.** Example of the search strategy for Medline (Ovid).

**Additional file 2: Table S2.** Study characteristics.

## Data Availability

The datasets used and/or analysed during the current study are available from the corresponding author on reasonable request.

## Abbreviations

AC1: First-order agreement coefficient
CI: Confidence interval
NICE: The National Institute for Health and Care Excellence
PROSPERO: The International Prospective Register of Systematic Reviews
