# Supplementary material for: Systematic review of the effect of training interventions on the skills of health professionals in promoting health behaviour, with meta-analysis of subsequent effects on patient health behaviours
Source: BMC Health Serv Res. 2020 Jun 29;20:593. doi: 10.1186/s12913-020-05420-1 (PMC7325030; doi:10.1186/s12913-020-05420-1)
Supplement: Supplementary file 1 — Additional file 1: Table S1. Example of the search strategy for Medline (Ovid). [file 12913_2020_5420_MOESM1_ESM.docx]

**Supplementary Table 1**

Example of the search strategy for Medline (Ovid).

| Search Terms for Medline (via Ovid MEDLINE(R) and In-Process & Other Non-Indexed Citations 1946 to March 04, 2019) | | | |
| --- | --- | --- | --- |
| 1 | Healthcare worker.ti,ab | **53** | Cross over.ti,ab |
| 2 | Physiotherapist.ti,ab | **54** | 45 or 46 or 47 or 48 or 49 or 50 or 51 or 52 or 53 |
| 3 | Nurs*.ti,ab | **55** | (Intervention ADJ3 fidelity).ti,ab |
| 4 | Exercise therapist.ti,ab. | **56** | (Quality ADJ3 delivery) .ti,ab |
| 5 | "General practitioner".ti,ab | **57** | (Performance ADJ3 improvement).ti,ab |
| 6 | Consultant.ti,ab | **58** | Skill*.ti,ab |
| 7 | Doctor.ti,ab | **59** | Professional ADJ3 practice.ti,ab |
| 8 | "Physical therapist".ti,ab | **60** | (Practice ADJ3 pattern*).ti,ab. |
| 9 | Dietitian.ti,ab | **61** | (Quality ADJ3 improvement).ti,ab. |
| 10 | Nutritionist.ti,ab | **62** | Competence.ti,ab |
| 11 | Physiologist.ti,ab | **63** | Fidelity.ti,ab |
| 12 | Physical scientist*.ti,ab. | **64** | (Intervention ADJ3 integrity).ti,ab. |
| 13 | "Rehabilitation engineer".ti,ab | **65** | (Intervention ADJ3 adherence).ti,ab |
| 14 | Health adviser.ti,ab | **66** | (Treatment ADJ3 integrity).ti,ab |
| 15 | Health trainer.ti,ab | **67** | (Intervention ADJ3 compliance).ti,ab |
| 16 | Physician.ti,ab | **68** | (Treatment ADJ3 compliance).ti,ab |
| 17 | Health professional*.ti,ab | **69** | (Intervention ADJ3 implementation).ti,ab |
| 18 | Health professions.ti,ab | **70** | (Treatment ADJ3 implementation).ti,ab |
| 19 | Health care worker.ti,ab | **71** | (Program ADJ3 integrity).ti,ab |
| 20 | Health care assistant.ti,ab | **72** | (Program ADJ3 adherence).ti,ab |
| 21 | Healthcare assistant.ti,ab | **73** | (Program ADJ3 compliance).ti,ab |
| 22 | Dietician.ti,ab. | **74** | (Program ADJ3 implementation).ti,ab |
| 23 | Matron.ti,ab. | **75** | (Treatment ADJ3 delivery).ti,ab |
| 24 | Therapist.ti,ab. | **76** | (Intervention ADJ3 delivery) .ti,ab |
| 25 | Community health service*.ti,ab. | **77** | (Program ADJ3 delivery).ti,ab |
| 26 | Practitioner.ti,ab. | **78** | 55 or 56 or 57 or 58 or 59 or 60 or 61 or 62 or 63 or 64 or 65 or 66 or 67 or 68 or 69 or 70 or 71 or 72 or 73 or 74 or 75 or 76 or 77 |
| 27 | 1 or 2 or 3 or 4 or 5 or 6 or 7 or 8 or 9 or 10 or 11 or 12 or 13 or 14 or 15 or 16 or 17 or 18 or 19 or 20 or 21 or 22 or 23 or 24 or 25 or 26 | **79** | Exercis*.ti,ab. |
| 28 | Training.ti,ab | **80** | Diet*. ti,ab |
| 29 | Teaching.ti,ab | **81** | Smoking.ti,ab |
| 30 | Learning.ti,ab | **82** | Alcohol.ti,ab |
| 31 | Simulation-based learning.ti,ab. | **83** | Selfcare.ti,ab |
| 32 | Seminars.ti,ab | **84** | Self care.ti,ab |
| 33 | Practice simulation.ti,ab | **85** | Weight.ti,ab |
| 34 | Workshops.ti,ab | **86** | Aerobic activ*.ti,ab |
| 35 | Collaboratives.ti,ab | **87** | Physical activ*.ti,ab |
| 36 | Train the trainer approach.ti,ab | **88** | Energy expenditure.ti,ab |
| 37 | Education.ti,ab | **89** | Health behaviour.ti,ab. |
| 38 | Mentoring.ti,ab | **90** | Health behavior.ti,ab |
| 39 | Online course*.ti,ab | **91** | Nicotine. ti,ab |
| 40 | Online module*.ti,ab | **92** | Drink*.ti,ab |
| 41 | Educational meeting.ti,ab | **93** | Sedentary. ti,ab |
| 42 | Simulation based training.ti,ab. | **94** | Run*. ti,ab |
| 43 | Self directed learning.ti,ab. | **95** | Swim*. ti,ab |
| 44 | 28 or 29 or 30 or 31 or 32 or 33 or 34 or 35 or 36 or 37 or 38 or 39 or 40 or 41 or 42 or 43 | **96** | Bicycl*.ti,ab |
| 45 | Trial*.ti,ab | **97** | Bicycl*.ti,ab |
| 46 | Randomi?ed.ti,ab | **98** | Cycl*.ti,ab |
| 47 | RCT.ti,ab | **99** | Walk*. ti,ab |
| 48 | Randomly. ti,ab | **100** | Jog*. ti,ab |
| 49 | (Singl* adj3 blind).ti,ab. | **101** | Danc*. ti,ab |
| 50 | Crossover.ti,ab | **102** | Gardening. ti,ab |
| 51 | Randomi?ation. ti,ab | **103** | 79 or 80 or 81 or 82 or 83 or 84 or 85 or 86 or 87 or 88 or 89 or 90 or 91 or 92 or 93 or 94 or 95 or 96 or 97 or 98 or 98 or 99 or 100 or 101 or 102 |
| 52 | Randomi?ed control*.ti,ab. | **104** | 27 AND 44 AND 54 AND 78 AND 103 |
