# Supplementary material for: Systematic review of the effect of training interventions on the skills of health professionals in promoting health behaviour, with meta-analysis of subsequent effects on patient health behaviours
Source: BMC Health Serv Res. 2020 Jun 29;20:593. doi: 10.1186/s12913-020-05420-1 (PMC7325030; doi:10.1186/s12913-020-05420-1)
Supplement: Supplementary file 2 — Additional file 2: Table S2. Study characteristics. [file 12913_2020_5420_MOESM2_ESM.docx]

**Supplementary table 2: Study characteristics**

| Study | Participants  (Design) | HCP training method and analysis method | HCP outcomes  (Communication style and delivery of intended intervention content, compared to control) | Effect on patient health behaviours | Percentage of significantly improved HCP outcomes |
| --- | --- | --- | --- | --- | --- |
| Brug *et al*, (21) | **HCPs:**  **Diabetes-care** **dietitians**  n= 37  (I= 18, C= 19)  Age: 24-45 years old  Gender: Not reported  **Patients: Homecare diabetes patients**  n= 142  (I= 83, C= 59)  Age and gender: Not reported  (RCT, cluster sampling - Nine home-care organisations, The Netherlands) | **Communication training:**  **Motivational interviewing training-** delivered by global motivational interview (MI) trainer and senior diabetes-care dietitian  **Intervention**: Two-days of MI training. First day: Introduction to theory and techniques.  Second day: Techniques were practiced  **Control**: Standard practice  **Follow-up time:** One-month and six months post intervention.  **Analysis:** Patient consultations were recorded and transcripts of the first 15-were minutes analysed.  The MI Skill Code 2.0 and  Motivational Interviewing Treatment Integrity code was used. | **Communication style**  **One-month post-training:**  Talk time(p=0.00), the total number of reflections (P=0.02) and MI infidelity (P=0.02) significantly increased. However, empathy, MI spirit, MI adherence, the number of closed and open questions, total questions, number of simple and complex reflections and number of change statements insignificantly increased (P>0.05)  **Six-months post-training:**  Talk time (p=0.00), empathy (p=0.01), number of simple reflections(p=0.01), MI spirt (p=0.01), MI infidelity (p=0.01), total number of reflections (p=0.01) and the number of times the patient changed a statement (p=0.01) significantly increased.  MI adherence, the number of closed and open questions, total questions and number of complex reflections insignificantly increased(P>0.05)  **Delivery of intended intervention content:** N/A | **Health behaviour change Intervention:** Diet counselling to improved diet.  **Primary outcome:** Saturated fat intake assessed via a seven-day food diary  **Patient follow-up:** Five-to-six months  **Overall effect:**  Saturated fat intake was lower compared to control; significance was not assessed (15.8 vs 18.4). | **One-month post-training**  **(short-term):**  Communication parameters: 25%  (3 of 12)  Delivery of intended intervention content parameters: N/A  Overall:25%  **Six months post intervention:**  Communication parameters: 58%  (7 of 12)  Delivery of intended intervention content parameters: N/A  Overall:58% |
| Casebeer *et al,* (22) | **HCPs:**  **Adult** **primary care physician** n= 28  (I= 14, C= 14)  Age and gender: Not reported  **Patients: Hyper- cholesterolemia patients**  n= 222  (I= 111, C= 111)  Age: >40 years old  Gender: 65.3% females  (RCT, cluster sampling - Primary care physicians’ clinics, Alabama) | **Job retraining:**  **Combined continuing medical education-** delivered by clinical psychologist and internist  **Intervention:** Three personalised interactive audio-conferences based on physicians’ knowledge and skill weaknesses.  Throughout, a strategy was developed, for physicians, to help increase adherence skills, chart reminders were provided  **Control**: Standard practice  **Follow-up time:** Three months  **Analysis:** Standardised patients assessed the number of behaviours used in accordance to the University of Rochester Risk Factor interview scale. | **Communication style:**  N/A  **Delivery of intended intervention conten**t**:** Overall, use of adherence enhancement methods and the overall number of adherence enhancement behaviours used insignificantly increased (P>0.05).  When considering adherence enhancement behaviours individually i.e. checking patient understanding of hypercholesterolemia,  risk factor assessment,  medication counselling and assessment, Interpersonal interaction, exercise counselling and diet counselling. Only checking patient understanding of hypercholesterolemia and Interpersonal interaction significantly increased (P=0.009 and P=0.028, respectively). | **Health behaviour change Intervention:** Diet counselling to improve management of hyper-cholesterolemia  **Primary outcome:** Serum cholesterol levels assessed via means standard lipid profile  **Patient follow-up:** Three/nine months  **Overall effect:** Three-months: Levels insignificantly improved (P>0.05).  Nine-months:  Levels for men significantly improved (P=0.02)  (control comparison) | **Three months post-intervention (short-term):**  Communication parameters: N/A  Delivery of trained content parameters: 25%  (2 of 8)  Overall:25%  (2 of 8) |
| Clark *et* al, (23) | **HCPs:**  **Paediatricians** n=74  (I= 38, C= 36)  Age: 30-60 years old  Gender:40% females  **Patients:**  Asthma child patients  n=701  (I= 336, C= 301)  Age: 1-12 years old  Gender: 30% females  (RCT, cluster sampling - Clinics, New York) | **Job retraining:**  **Retraining of current role based on National Asthma Education and Prevention Program**- delivered by asthma specialist  **Intervention:** Two 2.5-hour group meetings over 2-3 weeks that focused on how to observe, evaluate and act upon patient problems often confronted in asthma selfcare. The intervention aimed to improve HCPs communication skills and rapport and was practical and educational.  **Control**: Standard practice  **Follow-up time:** 48-months  **Analysis:** Assessed from practitioners’ point-of-view via a self-report questionnaire and from parents’ point of view via phone interview. | **Communication style:**  **From HCPs point-of-view:**  “The writing of ways for patients to adjust medication if their symptoms changed” and “providing methods for patients to adjust medication if their symptoms changed”, insignificantly increased (P=0.05 and P=0.06, respectively).  **From the parents’ point-of-view:**  All communication parameters improved significantly, parents reported HCPs: paid attention to family thoughts (P=0.037), made medication instructions easy to follow (P=0.004), inform them of short-term treatment plans (P=0.032), considered and talked about parents’ concerns with medication (P=0.018), increased number of open questions (P=0.031) and let them know they did the correct thing (P=0.022).  **Delivery of intended intervention content: From HCPs point-of-view:** Use of protocol to track education significantly increased (P=0.02).  **From the parents’ point-of-view:**  Time spent with patient insignificantly improved (P=0.067) | **Health behaviour change Intervention:** Asthma self-care programme to aid asthma self-management.  **Primary outcome:** Frequency of hospital admissions in last 12-months i.e. hospitalisation, emergency department visits, scheduled visits and follow-up visits- via physician office records  **Patient follow-up:** 12-months  **Overall effect:** Compared to control hospitalisations significantly decreased (P=0.03) | **48-months post-intervention (long-term):**  Communication parameters: 75%  (6 of 8)  Delivery of intended intervention content parameters: 50%  (1 of 2)  Overall: 70%  (7 of 10) |
| Cooper *et* al, (24) | **HCPs:**  **Physicians**  n=41  (I= 22, C= 19)  Age: 43 years old (mean)  Gender: 52% female  **Patients: General patients of physicians** n=279  (I= 84, C= 55)  Age: 61.3 years old (mean)  Gender: 52% female  (Two-by-two factorial RCT, stratified cluster sampling-  14-urban community- primary care sites, Maryland, USA) | **Communication training:**  **Communication skills**  **training-** delivered by community health workers  **Intervention:** 30-minute coaching session on how to communicate effectively with patients. Sessions were personalised based on physician’s interaction with a standardised patient at baseline. Five-behaviours linked with good hypertension management were taught.  **Control**: Standard practice (Given treatment guidelines and a newsletter)  **Follow-up time:** 12 months  **Analysis:** Consultations were recorded. Use of parameters were analysed, in accordance to the Roter Interaction Analysis System. Patient feedback via interview was also used. | **Communication style:**  **Consultation recordings:**  Physicians significantly improved “physicians’ patient-centeredness ratio\|” with standardised patients (P=0.02) and with actual patients (P=0.04). However verbal dominance ratio insignificantly increased with both standardised and actual patients (P>0.05).  **Patient feedback:**  “Physicians’ Participatory Decision-Making” and “Patient involvement in care”, was reported as insignificantly increasing (P=0.12 and P=0.14, respectively). Information exchange and patient decision making also insignificantly increased (both at P=0.14)  **Delivery of intended intervention content:** N/A | **Health behaviour change Intervention:** General health behaviour counselling  **Primary outcome:** Systolic blood pressure- assessed via an automatic oscillometric monitor  **Patient follow-up:** 12-months  **Overall effect:**  Compared to control after 12-months no significant changes occurred (P>0.05). | **12-months post-intervention (long-term):**  Communication parameters: 25%  (2 of 8)  Delivery of intended intervention content: N/A  Overall: 25%  (2 of 8) |
| De Ruijter  *et* al, (25) | **HCPs:**  **Practice nurses** n= 297  (I= 147, C= 122)  Age: 23-66 years old  Gender: 97.8% female  **Patients:**  **Smokers**  n= 391  (I=217, C=174)  Age and gender: Not reported  (RCT, cluster sampling - Health care clinic, The Netherlands) | **Job retraining:**  **Tailored e-learning program-** self-administered  **Intervention:** Based on nine STIMEDIC counselling steps via an e-learning programme for six-months. Elements included were: 1) counselling materials for smoking cessation, 2) tailored advice (based on: the I-change model, cessation guidelines and motivational factors and behaviour), 3) a forum, 4) a counselling checklist, 5) information about the study.  **Control:** ‘General’ modules only  **Follow-up time:** Six-months  **Analysis:** Overall guideline adherence and individual STIMEDIC guideline adherence was assessed. Adherence was assessed via an online self-report questionnaire. Post counselling sessions nurses also completed a checklist. | **Communication style**  N/A  **Delivery of intended intervention content:**  **Nurses with counselling experience:**  Overall use of guidelines was significantly improved (>9-years’ experience) (p=0.048).  **Nurses limited of counselling experience:** Overall, use of guidelines was insignificantly increased (p>0.05).  **For all nurses:**  Regarding adherence to the nine- STIMEDIC guidelines individually i.e. Advise of smoke quitting, assessment of smoking history, assessment of quit motivation, increasing motivation, assessment of quitting barriers, discussing of barriers, providing informing about cessation aids, designing a quit plan with a quit date, and arranging a follow-up post-quit date. Only assessment of quit motivation significantly increased (P=0.03) | **Health behaviour change**  **Intervention:** Smoking cessation counselling  **Overall effect:**  Not assessed | **Six-months post-intervention (medium-term):**  **Nurses with experience:**  Communication parameters: N/A  Delivery of intended intervention content parameters: 20%  (2 of 10)  Overall: 20%  (2 of 10)  **Nurses limited of experience:**  Communication parameters: N/A  Delivery of trained content parameters: 10%  (1 of 10)  Overall: 10%  (1 of 10) |
| El-Sayed  *et* al, (26) | **HCPs:**  **Physicians**  n= 40  (I= 20, C= 20)  Age and gender: Not reported  **Patients:**  Mother-child pairs n=480  (I= 240, C= 240)  Age: I= 11.8 months old,  C= 12 months old (means)  Gender: I=51.2% female, C=52.5% female  (RCT, cluster sampling- Primary health centre ,Egypt) | **Job retraining:**  **World health organisation (WHO) complementary feeding counselling-** delivered by five WHO experts  **Intervention:** Three-day course which emphasised on improving the knowledge and counselling skills of complementary feeding. The course involved theoretical and practical sessions; ongoing feedback was also given by master trainers.  **Control:** Less intensive management of childhood illness course  **Follow-up time:** Six-months  **Analysis:** Consultations were observed by trainers and patients (mothers). Communication parameters used were assessed in accordance to the complementary feeding counselling training course. | **Communication style:**  Significant improvements in; praise given to mother, amount of practical help given, use of self-help cards, simple language within counselling and checking mothers understanding (P<0.05).  However, use of acceptance statements insignificantly increased (P>0.05).  **Delivery of intended intervention content:** 22 of the 28-parameters significantly increased (P<0.05) i.e. Physician reported more about: quantity consumed, mode of feeding, food consistency, amount of food, food preparation, ask/advice eating habits (meal and snack amount and food type; dairy, cereals, greens, protein), ask about food type (thickness, cereals, greens, protein, feed frequency, proper portion size), telling mother to provide encouragement and habits in illness. Insignificantly (P>0.05) improved parameters included: Asking if: child breastfeeds, frequency breastfed day and night, breastfeeding two-years, complementary feeding after six-months and giving follow-up dates | **Health behaviour change Intervention:** Diet counselling to improve mother feeding behaviour  **Primary outcome:**  Child weight gain  **Patient follow-up:** Six-months  **Overall effect:**  Compared to control mean weight gain significantly increased (P=0.02) | **Six-months post- intervention (medium-term)**:  Communication parameters: 83%  (5 of 6)  Delivery of intended intervention content parameters: 79%  (22 of 28)  Overall: 79%  (27 of 34) |
| Flocke *et* al, (27) | **HCPs:**  **Community-based primary care clinicians** n=31  (I= 16, C= 15)  Age: 47 years old (mean)  Gender: 48% female  **Patients:**  **Smokers**  n=840  (I= 407, C= 402)  Age: 44 years old (mean)  Gender: I=58%, C=63% female  (Group randomized Clinical trial, stratified cluster sampling- Practice clinic, United states) | **Communication training:**  **Teachable moment communication process (TMCP)**- delivered by instructor  **Intervention:** Two, 3-hour educational training sessions on TMCP elements, involving: a presentation, video skill demonstrations, and practicing with standardized patients. Feedback from peers and trainers was given throughout.  **Control:** A multimedia education resource for colon  cancer screening  **Follow-up time:** Average 35-days (one-month)  **Analysis:** Consultations were recorded, the number of communication parameters used was assessed against the TMCP elements. | **Communication style:**  Physicians significantly increased linking smoking to patients concerns (P=0.01) and use of four brief advice elements i.e. expressing concern (P=0.007), optimism (P=0.001), partnership (P=0.003) and engagement in open conversations about patients thoughts on quitting (P=0.006).  Additionally, overall alignment of patient’s readiness to quit with clinician’s communication performance (P=<0.001), the duration of smoking discussion (P=0.01) and proportion of visits that smoking was discussed in, significantly increased (P=0.01). Physicians were also more likely to provide advice that aligned with patient’s level of readiness (P=<0.001). However, the use of quit statements increased insignificantly (P>0.50).  **Delivery of intended intervention content:** N/A | **Health behaviour change Intervention:** Smoking cessation counselling to aid quit readiness  **Primary outcome:**  Patient readiness to quit- assessed via patient report scale: ‘not ready, ‘ambivalent’, ‘ready’ or ‘unclear/ unknown’ via interview  **Patient follow-up:** Average 35-days (one-month)  **Overall effect:**  Compared to control, patients were more likely to report as ‘ready to quit’ (65% vs 84% P=0.006). | **35-days (One-month) post-intervention (short-term):**  Communication parameters: 90%  (9 of 10)  Delivery of intended intervention content parameters: N/A  Overall: 90%  (9 of 10) |
| Karvinen  *et al,* (28) | **HCPs:**  **Oncology nurses**  n=54  (I= 24, C=24)  Age: 45.4 years old (mean)  Gender: 98% female  **Patients:** N/A  (Related to cancer patients)  (RCT, cluster sampling - Online, Canada or United states) | **Job retraining:**  **Social cognitive theory based physical activity learning modules**- delivered online.  **Intervention:** Six modules, over 12-weeks based on: motivational interviewing, physical activity benefits and guidelines in cancer survivorship, barriers to physical activity counselling, keeping active strategies and behaviour change motivational strategies. Evaluation of case studies, videos and a quiz were in each module.  **Control:** Standard practice (given a list cancer websites).  **Follow-up time:** Instantly  **Analysis:** Questionnaire and  Likert scales | **Communication style:** N/A  **Delivery of intended intervention content:** Nurses significantly improved their adherence to Physical activity counselling methods due to significantly improved self-efficacy for counselling (P=0.007) and understanding of the barriers to counselling (P=0.013). However, three parameters: Counselling amount, knowledge of physical activity for cancers survivors and counselling’s perceived benefits insignificantly increased (P>0.05). | **Health behaviour change Intervention:** Physical activity counselling  **Overall effect:**  Not assessed | **Instantly post- intervention (short-term)**:  Communication parameters: N/A  Delivery of intended intervention content parameters: 40%  (2 of 5)  Overall: 40%  (2 of 5) |
| Kinmonth  *et* al, (29) | **HCPs: Nurses/GPs**  n=107 (I= 55, including 23 GPs and 32 nurses. C= 52 including 20 GPs and 32 nurses).  Age and gender: Not reported  **Patients:**  **Type-two diabetes patients**  n=360  (I=199, C=161)  Age: 57 years old (mean)  Gender: 31% female  (RCT, stratified cluster sampling - Practice clinics, southern England) | **Job retraining:**  **Retraining based on national diabetes guidelines and materials with additional Patient centred counselling training-** delivered by experienced facilitator  **Intervention:**  First day: Half-a-day reviewing evidence for patient centred counselling followed by training of negotiating behaviour change and active listening skills.  Second day: Full day of practicing taught skills (GPs only performed part-one). HCPs also received a diabetes information booklet on behaviour change and a leaflet encouraging patients to share concerns, to give to their patients.  **Control:** Standard practice with guidelines  **Follow-up time:** 12-months  **Analysis:** Patients/GP rated HCPs communication via a scale (0-6 range, 6= always report personal issues) | **Communication style:** Following training, GPs had significantly more maximum communication scores from patients (P=0.001), showing improved patient centred counselling skills. Nurses maximum communication scores from patients, improved insignificantly (P=0.12).  **Delivery of intended intervention content:** N/A | **Health behaviour change Intervention:** Diet counselling to improve diabetes self- care behaviour  **Primary outcome:**  HaemogoblinA_1C_% - assessed via haemoglobin A_1c_ion exchange  chromatography  **Patient follow-up:** 12-months  **Overall effect:** After 12-months, compared to control, HaemogoblinA_1C_% decreased insignificantly (7.07 vs 7.17, P=0.31). | **12-months post- intervention (long-term):**  Communication parameters: 50%  (1 of 2)  Delivery of intended intervention content parameters: N/A  Overall: 50%  (1 of 2) |
| Moore *et al,* (30) | **HCPs:**  **Practitioners**  n=84  (I=42, C=42)  Age: Not reported  Gender: I= 86% female, C= 84%, female  **Patients:**  **General patients of physicians** n=2400  (I=1200, C=1200)  Age: I= 49 years old, C= 44 years old (mean)  Gender: I=50% female, C= 62%, female  (RCT, stratified cluster sampling - general practices, Sunderland) | **Job retraining:**  **Nutritional Education training-** delivered by clinical dietitian  **Intervention:** A two phased programme over six-months. Phase-one: New skills were highlighted (i.e. patient assessment, education and goal setting techniques) over three 90-minute sessions.  Phase-two: HCPs practiced the techniques over two 90-minute sessions.  **Control:** Standard practice (no additional training)  **Follow-up time:** Instantly  **Analysis:** Patient questionnaires developed within study, assessed practitioner behaviour. A case study to assess application of knowledge | **Communication style:** Regarding the patient responses no communication element significantly increased i.e. Asking about food eaten, asking to change eating habits, advice was helpful, involvement in decisions, food specific advice, fruit and vegetable intake increase advice, (P>0.05).  **Delivery of intended intervention content:** The case study showed that, trained practitioners were significantly better at providing appropriate advice (P<0.01), but asking appropriate dietary related questions (P=0.84) insignificantly increased  Additionally, following training, giving of leaflets by HCPs significantly increased (P=0.004). | **Health behaviour change Intervention:** Diet counselling  **Overall effect:**  Not assessed | **Instantly** **post- intervention (short-term)**:  Communication parameters: 0%  (0 of 6)  Delivery of intended content: 67%  (2 of 3)  Overall: 22%  (2 of 9) |
| Pelto *et al,* (31) | **HCPs:**  **Physicians**  n=31  (I=17, C=14) from 28 municipal health care centres  Age and Gender: Not reported  **Patients:**  **Caregivers/child pairs (home visits)**  n= 424  Age: Child <1-months old  Gender: N/A  and  Nutrition sub-study  **Caregivers/child pairs**  n=69  (I= 37, C=32)  Age: Child <1-months old  (RCT, stratified cluster sampling - municipal health centres, Brazil) | **Job retraining:**  **Integrated Management of Childhood Illness (IMCI) nutrition counselling module training-** delivered by experienced facilitator  **Intervention:** 20-hours of nutritional counselling education training. Basic but up-to-date knowledge and practical counselling methods on nutrition were taught. HCPs then practiced the new techniques.  **Control:** Received minimal clinical refresher course  **Follow-up time:** Six-months  **Analysis:** Consultations (home visits) were observed. A checklist assessed the use of; physician nutritional advice given, description of clinical examination procedures and communication skills. | **Communication style:**  Giving of praise only or checking understanding only, significantly increased (P=0.01 and P=0.04, respectively). Giving both praise and checking understanding also significantly increased (P=0.01). Additionally, giving neither praise nor checking understanding significantly decreased (P=0.01).  **Delivery of intended intervention content:** Regarding giving nutritional advice; the total number of nutritional advice messages given significantly increased (P=0.014). Likewise , the amount of consultations with 0 advice messages given significantly decreased (P=0.02). | **Health behaviour change Intervention:** Diet counselling to improve nutritional knowledge for self-care  **Primary outcome:**  Nutrition messages recalled: assessed via interview  **Patient follow-up:**  One-week  **Overall effect:** The total number of nutritional advice messages recalled by patients significantly increased compared to control (P=0.01). Specifically, messages recalled regarding specific foods and feeding practices significantly increased (P=0.01). However, messages recalled regarding breastfeeding insignificantly increased (P>0.05). | **Six-months post- intervention (medium-term)**:  Communication parameters: 100%  (4 of 4)  Delivery of intended intervention content parameters: 100%  (2 of 2)  Overall: 100%  (6 of 6) |
| Presseau  *et al,* (32) | **HCPs:**  **GPs/ healthcare assistants** n=312  (I= 140, including 64 GPs, 29 Nurses, 20 Healthcare assistants and 17 “other”. C=172 including 87 GPs, 54 Nurses, 31 Healthcare assistants).  Age and Gender: Not reported  **Patients:**  Type-two diabetes patients **n= 4085**  (I=2110, C=1975)  Age: <18 (18% <65)  Gender: Not reported  (RCT, cluster sampling - General practices, North-east England) | **Job retraining:**  **Improving Diabetes care through Examining, Advising and prescribing trial (TIDieR)-** delivered by expert  **Intervention:** 90-minute workshop involving: presentations, demonstrations, discussions, group/individual work and watching videos of case studies. Online reading was available as well as help sheets healthy eating and exercise advice  **Control:** Standard practice (no additional training)  **Follow-up time:** 12-months  **Analysis:** Assessment of prescribing for glycaemic and blood pressure control and foot blood circulation examination was performed using patient records. Assessing advice given on nutrition, exercise and diabetes education was performed via patient survey. | **Communication style:**  N/A  **Delivery of intended intervention content:** Post intervention, the practice of ensuring/checking blood circulation in type-two patients’ feet significantly increased compared to control (P=0.01). However additional prescribing of therapy for glycaemic control and blood pressure control insignificantly increase (P=0.13 and P=0.29, respectively). Regarding the giving of advice: giving type-two diabetes education significantly increased (P=0.03), giving personalised nutrition and exercise advice insignificantly increased (P=0.95 and P=0.17, respectively. | **Health behaviour change Intervention:** Diet counselling  **Overall effect:**  Not assessed | **12-months post- intervention (long-term):**  Communication parameters: N/A  Delivery of intended intervention content parameters: 33%  (2 of 6)  Overall: 33%  (2 of 6) |
